# Development of ultra-thin radiation-shielding paper through nanofiber modeling of morpho butterfly wing structure

**DOI:** 10.1038/s41598-022-27174-y

**Published:** 2022-12-29

**Authors:** Seon-Chil Kim, Hongsik Byun

**Affiliations:** 1 Department of Biomedical Engineering, Keimyung University School of Medicine, Daegu, Korea; 2 Department of Chemical Engineering, Keimyung University, Daegu, Korea

**Keywords:** Biomedical engineering, Chemical engineering

## Abstract

In medical institutions, radiation shielding is an effective strategy to protect medical personnel and patients from exposure. Reducing the weight of the shield worn by medical personnel in the radiation generating area plays a key role in improving their productivity and mobility. In this study, a new lightweight radiation shield was developed by electrospinning a polymer-tungsten composite material to produce nanofibers with a multi-layered thin-film structure similar to that of a morpho butterfly wing. The fabricated shield was in the form of 0.1 mm thick flexible shielding paper. The multi-layer structure of the thin shielding paper was obtained through nanofiber pattern formation via electrospinning a dispersion of tungsten particles. At 0.1 mm thickness, the paper’s shielding rate was 64.88% at 60 keV. Furthermore, at 0.3 mm thick and arranged in a laminated structure, the shielding rate was 90.10% and the lead equivalent was 0.296 mmPb. When used as an apron material, the weight can be reduced by 45% compared to existing lead products. In addition, the material is highly processable and can be used to manufacture various flexible products, such as hats, gloves, underwear, and scarves used in medical institutions.

## Introduction

Radiography is a medical technology that transmits X-rays through the human body and it uses the difference in the density of substances in the human body to image anatomical structures^1^. The penetration of X-rays is limited when the density of the tissue is high, whereas tissues with relatively low density can be easily penetrated^2^. Therefore, the higher the density of the shield, the more advantageous it can be for radiation protection.

Artificial radiations, such as X-rays, have been developed for medical and industrial technologies. However, owing to the increasing use of medical devices, the general population and medical and industry workers are subjected to increased radiation exposure^3^. Therefore, active radiation defense technology is required to reduce exposure. Furthermore, the use of mobile X-ray devices has increased because of the recent COVID-19 pandemic^4^. The International Commission on Radiological Protection (ICRP) specifies that radiation used in the medical field should be used for the benefit of patients and should be optimized^5^.

Radiation shield technology used in medical institutions is associated with time and distance^6^. Lead plates or sheets made of lead powder and a polymer, such as rubber, are generally used as X-ray shields^7^. However, owing to its toxicity, lead poses problems with lead poisoning and disposal. Therefore, shields used in medical institutions are increasingly being manufactured with lead-free materials^8^. However, most medical devices, supplies, and facilities that use radiation still use lead as a shielding material. Therefore, to overcome this issue, the use of cheap and eco-friendly lead-free materials with shielding performance equivalent to lead should be expanded.

Materials such as tungsten, bismuth oxide, barium sulfate, and boron are typically used as alternatives to lead^9^. Considering the shielding performance, tungsten is the most useful eco-friendly shielding material. In general, lead replacement shielding materials should be non-toxic and have flexibility and processability. In addition, the materials should be proposed as a material having an excellent affinity with the polymer to be mixed or as a material capable of reducing the weight when manufacturing a shield. The types of shields that can be produced with these shielding materials include plate, fiber, and sheet, and pressing or injection molding into the desired shape is possible depending on the process technology.

A fiber-type shield is woven from a yarn impregnated with the shielding material. However, the shielding performance is limited by the pinholes generated between the yarn during the weaving process. Therefore, fiber-based shields are used primarily to protect against secondary (or scattered) radiation^10^. A sheet-shaped shield is manufactured by mixing a polymer and shielding material, which is compressed to the required thickness. The most important element of this process is the uniform dispersion of the shielding material. The shielding material dispersion process affects the reproducibility of the shielding performance and is difficult to apply to mass production without standardization of the production process^11^.

The single component plate-shaped shield comprises 100% of the shielding material and is manufactured through the rolling process. When tungsten is selected as the shielding material during single component plate manufacturing, the production processability is low because of its high melting point^12^. Therefore, the choice of shielding material for single component plate shields is limited. In recent years, plate flexibility has been obtained by using composite materials and the plate manufactured in this manner has been widely used as a material for applications such as shielding walls^13^. Materials for other applications of shields, such as blocks, syringe shields, and apertures used in medical institutions, are manufactured by injection molding by mixing a shielding material and polymer^14^. The miscibility of the metal particles with the polymer is a crucial factor affecting processability and shielding performance of composite materials.

An apron is a representative medical institution X-ray shield, which is manufactured in the form of clothing and worn by medical staff and workers. Therefore, it must be manufactured in a thin and light form to ensure unconstrained mobility of the wearer. The X-ray shielding apron currently available places a physical burden on the wearer because it weighs 2.85–3.15 kg for a product with a lead equivalent of 0.25 mmPb^15^. The weight reduction of the shielding garment may be limited because it is directly related to the density and mass of the shielding material. Although the mobility of the wearer can be improved by reducing the thickness of the sheet, this would reduce the shielding performance. A method to improve the shielding performance is the controlled dispersion of the shielding material. The attenuation of the incident X-ray energy occurs by its interaction with shielding material particles, which can be increased by allowing the radiation to interact with a greater number of particles^16^. Therefore, the particle dispersion technology of the shielding material is the most crucial factor in terms of shielding performance, weight reduction, and reproducibility of shielding performance, especially for the thin layer composite structures.

Several types of unusual composite structures are found in nature. The wings of the morpho butterfly are composed of micro-sized multi-layered thin films and show a regular arrangement. Due to the unique surface structure, only blue light is reflected and the butterfly wings appear blue^17^. The wrinkles are folded on the left and right pillars with an interval of approximately 700 nm and a height of 2 μm, and the interval between the upper and lower wrinkles is approximately 200 nm^18^. In this study, the structure of butterfly wings was used as a model for the dispersion of particles in an X-ray shield. The pattern was completed by repeated overlapping, similar to the tile used for the roof of a Korean building. In addition, to maintain the reproducibility of this pattern, the electrospinning method was used to apply the same amount of shielding material at the same location. The shielding material was eco-friendly tungsten powder particles. Even though tungsten has an atomic number of 74 and has a density higher than that of lead (19.25 g cm^−3^), a weight reduction is possible by reducing the thickness of the shield^19^. Therefore, this study aims to evaluate the shielding performance of a shield with a pattern similar to butterfly wings based on these materials.

In addition, this study aims to improve the shielding performance by making the radiation shielding paper as thin as possible so that it can be used in a multi-layered structure, and inducing interaction with particle radiation. The shielding paper produced can be used in a flexible, laminated structure because of its reduced thickness. Thus, this study reports a novel medical radiation technology for manufacturing thin shielding papers utilizing the morpho butterfly wing structure. Moreover, the proposed method can be used to mass produce the lightweight radiation-shielding paper and improve the safety of medical workers.

## Methods

Radiation should be increased. The particle dispersion technology of the shielding material can improve the shielding performance by increasing these interactions. Therefore, for a medium composed of polymer and tungsten particles, the reduction in the intensity of the incident energy by its interaction with a mass per unit area of the shielding material can be calculated by the Beer–Lambert Equation^20^:

1 $$ \begin{array}{*{20}c} {I = I_{0} e^{ - \mu d} } \\ \end{array} $$

where ${I}_{0}$ is the incident photon intensity, $I$ is the attenuated photon intensity, $\rho$ (g cm^−3^) is the density, and $\mu$(cm^−1^) and ${\mu }_{m}$(cm^2^ g^−1^) are the linear and mass attenuation coefficients, respectively. The thickness of the shield, $d$(cm), corresponds to the distance at which the incident ray interacts with the particles of the shielding material. Therefore, ${d}_{m}$ (g cm^−2^) is the mass per unit area of the shielding paper, and when this is calculated as the thickness of the shield, it can be expressed as Eqs. (2), (3):

2 $$ \begin{array}{*{20}c} {\mu_{m} = \frac{\mu }{\rho } = \frac{{I_{0} /I}}{\rho d} = \frac{{I_{0} /I}}{{\mu_{m} }}} \\ \end{array} $$

3 $$ \begin{array}{*{20}c} {d_{m} = \left( {\frac{\mu }{\rho }} \right) = \mathop \sum \limits_{i} W_{i} \left( {\frac{\mu }{\rho }} \right)_{i} } \\ \end{array} $$

where ${W}_{i}$ is the weight ratio of the $i$th component^21^. This implies that the number of shielding materials in the radiation shield must be higher to increase the mass per unit area. Therefore, to improve the shielding effect with a minimum increase in mass, the unit area of the shielding material must be increased. The total atomic cross-sectional area ${\sigma }_{a}$(cm^2^ g^−1^) of the shielding paper can be estimated using the mass attenuation coefficient. By calculating the number of atoms, the electron density can be obtained as:

4 $$ \begin{array}{*{20}c} {d_{m} = \frac{{N_{A} \sigma_{a} }}{N}} \\ \end{array} $$

where ${N}_{A}$ is the Avogadro constant. Therefore, the electron cross-sectional area can be obtained as:

5 $$ \begin{array}{*{20}c} {d_{m} = \frac{1}{{N_{A} }}\left[ {\mathop \sum \limits_{i} \frac{{f_{i} A_{i} }}{{Z_{i} }}(\mu_{m} )_{i} } \right]} \\ \end{array} $$

where ${Z}_{i}$, ${f}_{i}$, $({\mu }_{m}{)}_{i}$, and ${A}_{i}$ are the atomic number, mole fraction, mass attenuation coefficient, and atomic weight of the *i*^th^ component, respectively^22^.

**Figure 1:**
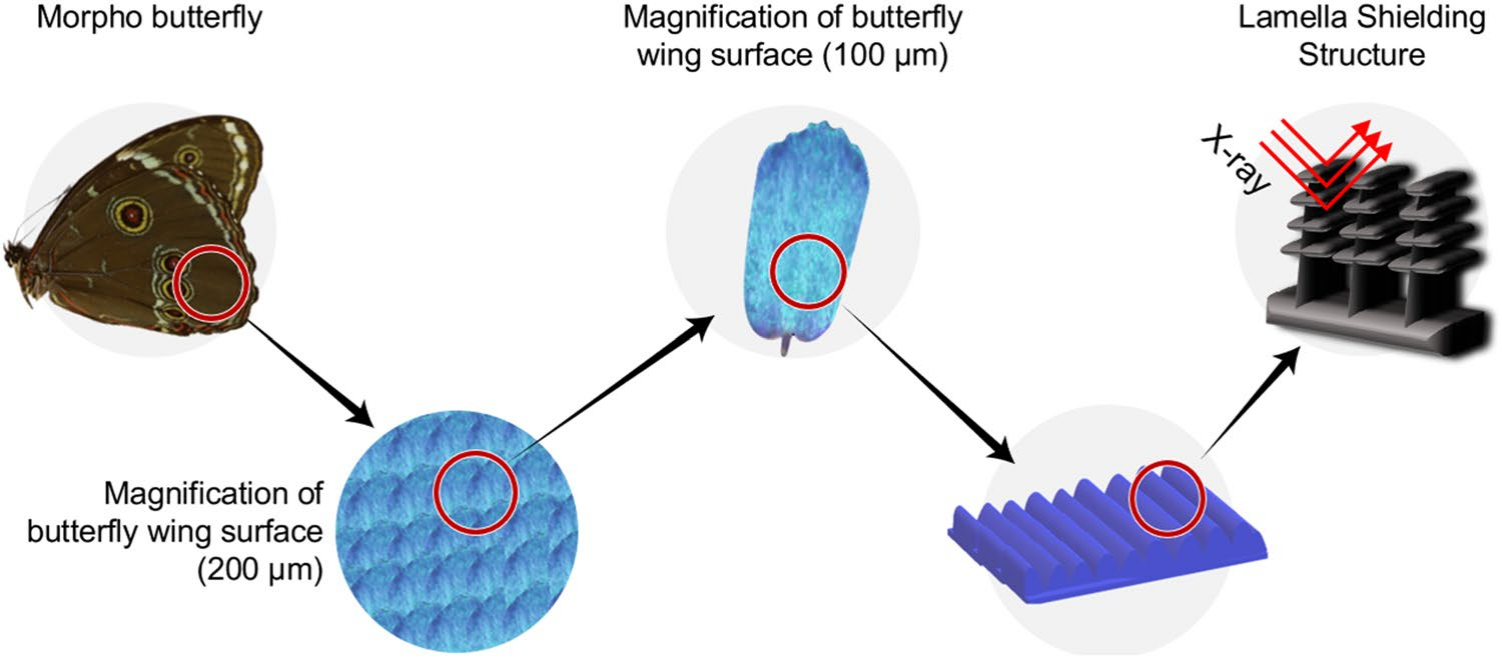
Magnified view of a morpho butterfly’s wing and its application to X-ray shielding.

To increase the dispersibility of the shielding material particles, a new dispersion structure was applied for producing the shielding paper. The dispersion of the shielding material was carried out to widen the total atomic cross-sectional area, and the morpho butterfly wing structure was selected as the most effective model. Figure 1 schematically shows the use of the multi-layered structure of the morpho butterfly’s wing for X-ray shielding.

**Figure 2:**
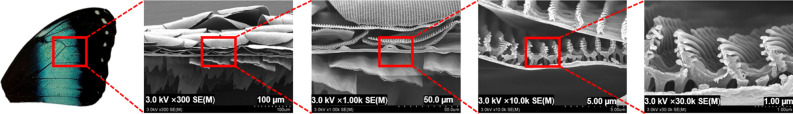
Enlarged structure of the morpho butterfly wing.

As shown in Fig. 2, the surface of the wings of the morpho butterfly is overlapping, and when viewed from the cross section, it can be seen that it has a multi-layered thin-film structure. Structurally, the thickness, the refractive index of the wing, and the periodicity of the grating are designed to reflect only blue wavelength^23^.

X-rays used in medical institutions have the characteristics of low turnover and are very straight. Therefore, if they are shielded with a multi-layered shield similar to a morpho butterfly wing structure, the cross-sectional area of the photon collision unit can be widened, and the shielding effect is expected to increase. Therefore, because the same pattern can be implemented through nanofibers, provided a tungsten shielding material can be grafted onto such a structure, structural dispersion can be used to improve the shielding performance.

**Figure 3:**
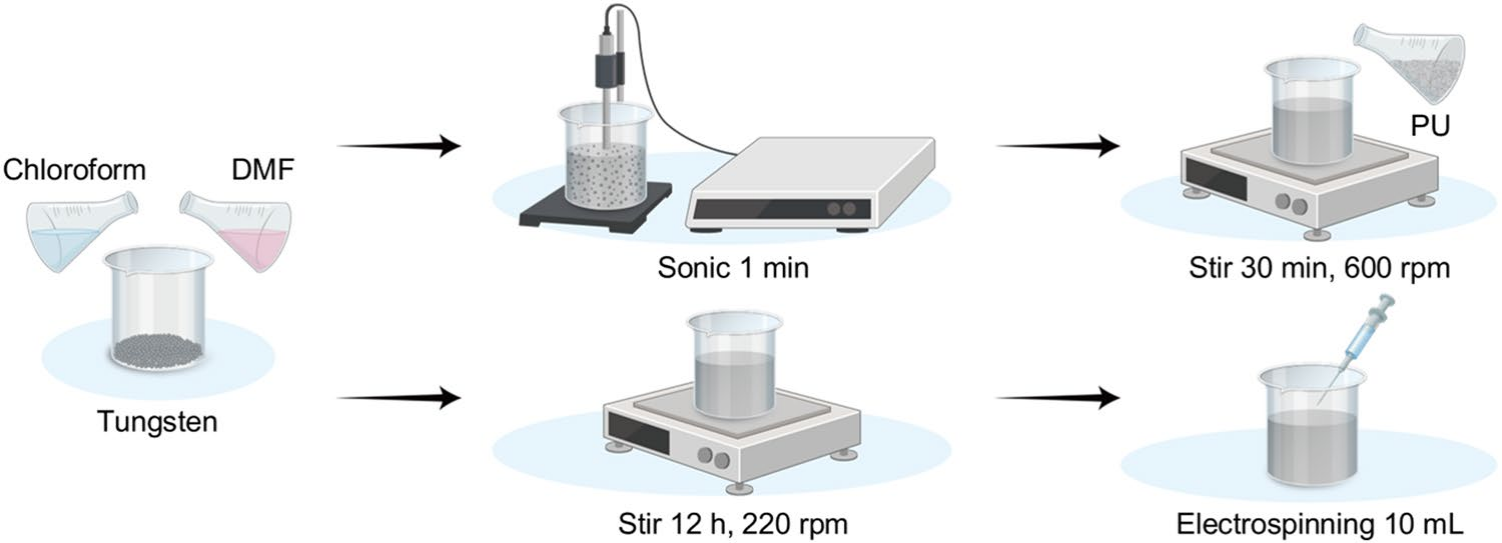
Preparation of the spinning solution used for the production of thin-film shielding paper.

The proposed shielding structure contains two constituent materials, tungsten and polyurethane. As shielding material, powdered tungsten (tungsten, W, 99.9%, < 4 µm, NanGong XinDun alloys spraying Co. Ltd., China) was used. The tungsten powder was crushed for 5 min and then dried in an oven at 60 °C for 24 h to control the particle size. The polymer used with tungsten was polyurethane (PU, P-7195A, M.W. 100,000–150,000, Songwon, Korea) that was dried under the same conditions as tungsten. N-dimethylformamide (DMF, 99.5%, Daejung, Korea) was used as a solvent to dissolve the polymer. Two solvents were used to prepare the shielding paper; chloroform (95%, Duksan, Korea) was used as a poor solvent to control the volatilization rate of the solvent, and DMF to dissolve the polymer. The preparation method of the spinning solution is shown in Fig. 3. First, the tungsten was placed in a 20 mL glass bottle. Subsequently, 5.165 g of DMF and 2.785 g of chloroform were added, dispersed for 1 min with an ultrasonic grinder, and mixed using a magnetic stirrer (Laboratory stirrer/hot plate, PC-420, Corning, Mexico) at 600 rpm. In addition, 2.05 g of PU was added and, after 10 min, the speed of the stirrer was reduced to 220 rpm, and the mixing was continued for 12 h or more until the polymer was completely dissolved and spun.

**Figure 4:**
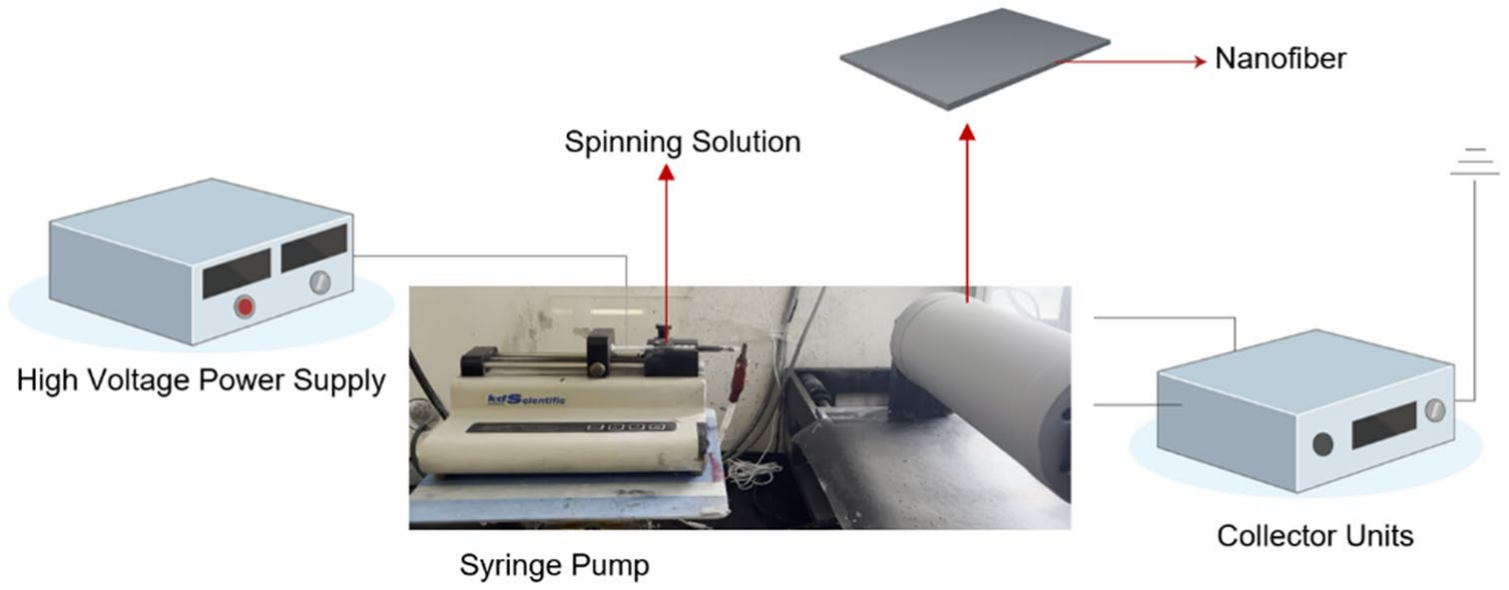
Electrospinning method to increase the dispersion power of the shielding material.

To increase the dispersion power of the shielding material, electrospinning was maintained at 10 kV by controlling the voltage with a high-voltage power supply (CPS-60K02VIT, Chungpa EMT Co., South Korea), as shown in Fig. 4. In addition, the spinning speed was adjusted so that the flow rate from a syringe pump (syringe pump, KDS100, SD Scientific Inc., Holliston, USA) was 1.0 mL h − 1. In this process, because the capacity and collection distance of the syringe affect the formation of the nanofiber pattern due to the particle weight of tungsten, the syringe was repeatedly moved to form the nanofiber pattern.

**Figure 5:**
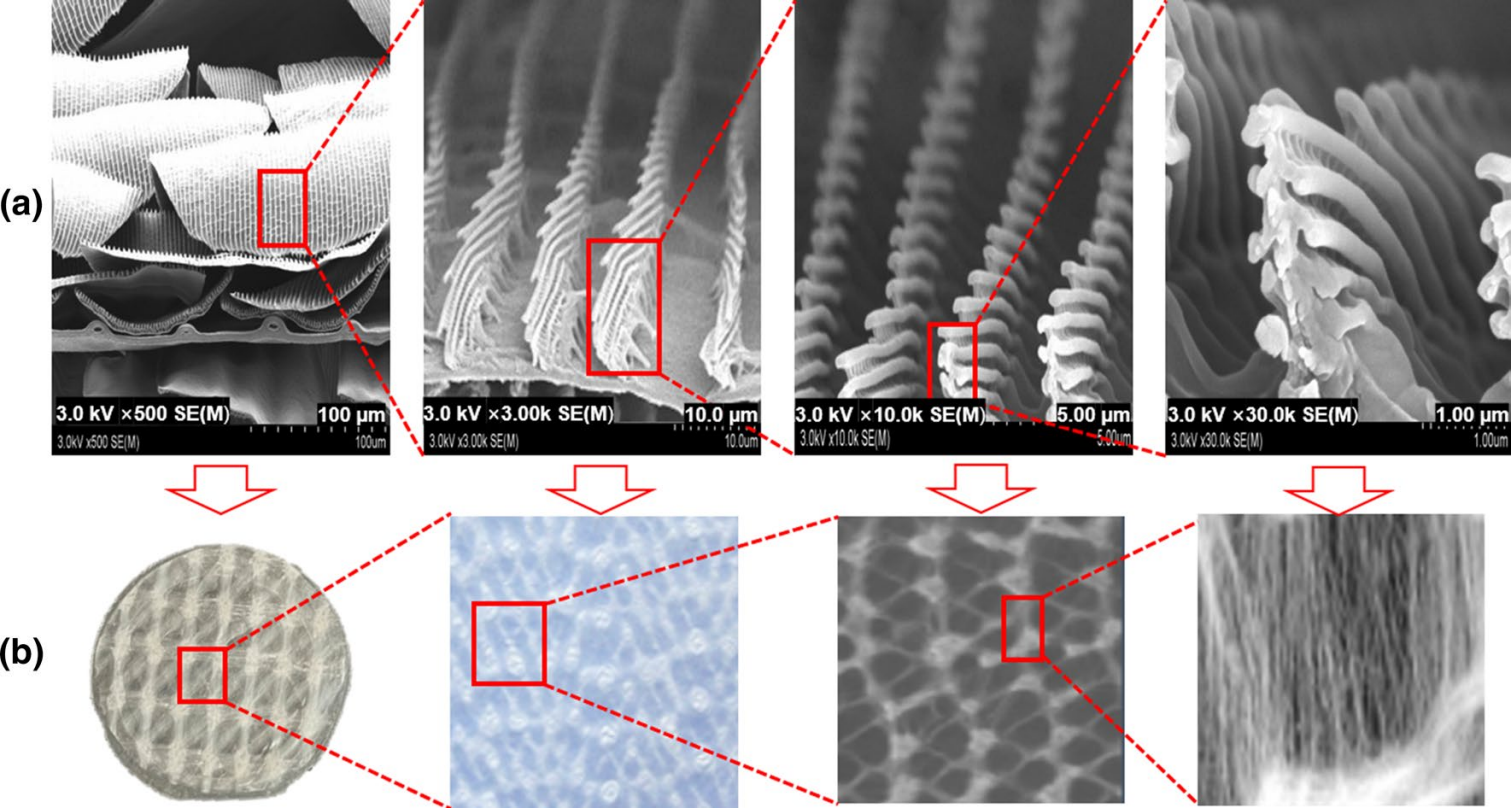
Electrospinning of the shielding material to increase its dispersion power. (**a**) Magnified images of a morpho butterfly wing, and (**b**) result of implementing the same pattern through electrospinning.

To reproduce the multi-layered structure of a morpho butterfly wing, an electrospinning technique was applied that maintained the same directionality. In general, nanofiber mats are produced by irregularly distributing the nanofibers without a fixed direction during the collection step^24,25^. However, if a nanofiber having a pattern structure in a certain and regular direction is manufactured, errors resulting from the irregular pattern structure can be reduced when radiation passes through the inner pattern of the nanofiber^26,27^^.^ In addition, if a regular nanofiber pattern is applied, the compactness inside the shield can be increased with the same amount of shielding material. As shown in Fig. 5, the structure of the butterfly wing (Fig. 5a) and the structure of the polymer pattern (Fig. 5b) were found to match.

The final shielding material dispersion conditions are shown in Table 1. Electrospinning was performed by maintaining the distance between the needle and the collecting plate at 13–15 cm, the humidity at 25–40%, and temperature at 22–25 °C. Furthermore, 10 mL of the electrospinning solution was electrospun at 1 h intervals of 1 mL each. Owing to the weight of the tungsten particles in the manufactured composite material, the shorter the time for electrospinning after stirring, the better the dispersion.

**Table 1:** Electrospinning condition for preparation of nanofibers.

Humidity (%)	Temperature (℃)	Voltage (kV)	TCD (cm)	Rate (mL h^−1^)	Duration (h)	Needle size (Gauge)
25–30	22–25	10	13–15	1.0	10	23

In addition, the nanofiber paper was subjected to a post-treatment process three times for 10 s using a heat press (heating press, DHP-2, Dad Heung Science, South Korea) at a temperature of 40 °C and a pressure of 3000 psi. This process was repeated five times to obtain a sheet of thin-film shielding paper with a thickness of 0.1 mm. The prepared thin-film shielding paper was observed with a field emission scanning electron microscope (FESEM; S-4800, Hitachi, Japan) to analyze the degree of dispersion^28^. Two different criteria were used for the observation, i.e., how well the particles the particles of the shielding material were dispersed and how close polymer pattern was to the structure of the morpho butterfly wing.

**Figure 6:**
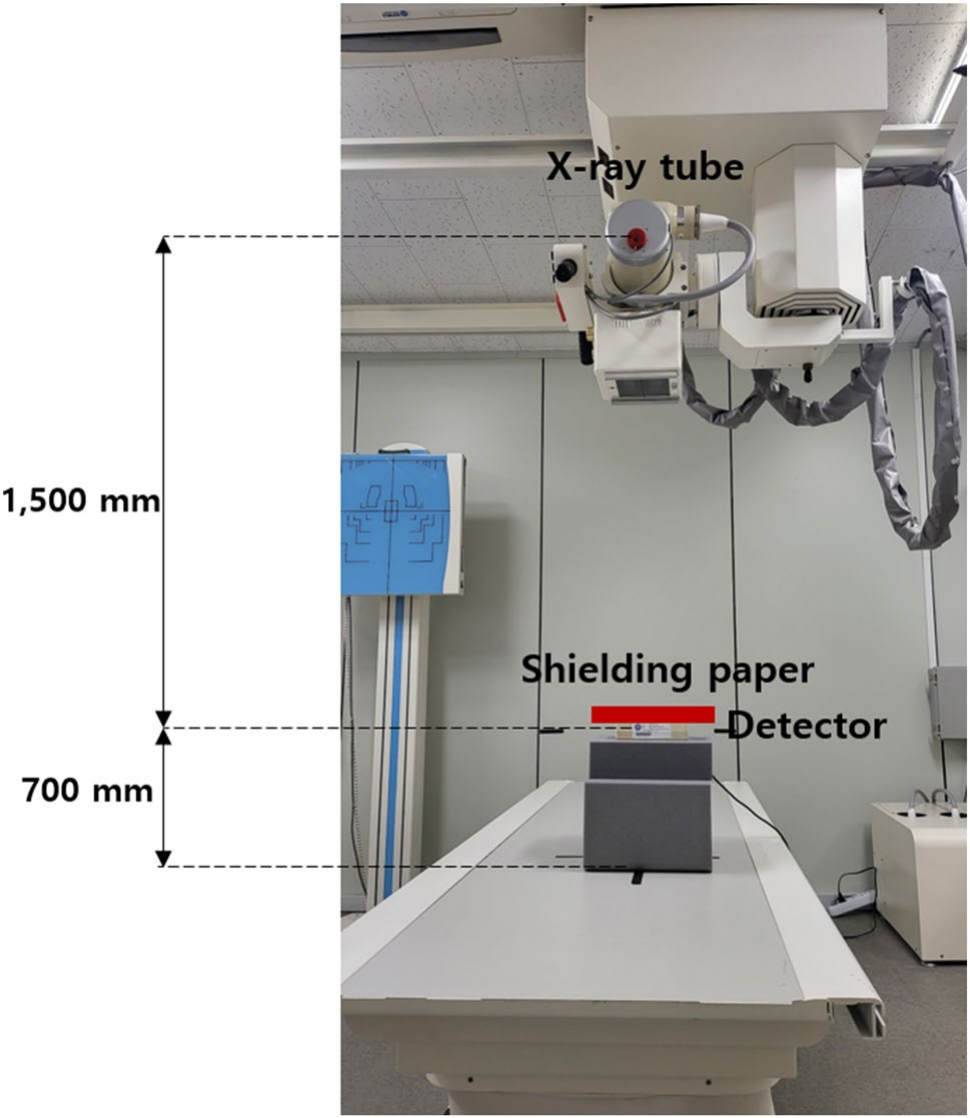
Experimental setup to evaluate the shielding performance of the shielding paper.

The evaluation of the shielding performance of the shielding paper was based on the geometric conditions shown in Fig. 6^29^. The medical radiation used in this experiment was converted into an effective energy, which is a single energy. Therefore, to measure the half-value layer (HVL), the slope was calculated from the attenuation coefficient law ($I={I}_{0}{e}^{-\mu x}$), and the linear attenuation coefficient $\mu$ was obtained from this slope, which was subsequently calculated from the HVL as 0.693/μ^30^. In addition, Hubbell’s mass attenuation coefficient table was used to calculate the effective energy that has the same value as the HVL corresponding to the single energy of the HVL obtained above^31^. The shielding rate of the shielding paper was calculated as $(1-\frac{W}{{W}_{0}})\times 100$^32^, where $W$ and ${W}_{0}$ are the doses measured with and without a shielding paper between the X-ray tube and the dosimeter, respectively. Moreover, the average of 10 measurements performed using an X-ray generator (Toshiba E7239, 150 kV–500 mA, 1999, Japan) was used for this purpose. The dose detector used an Ion Chamber (Model PM-30, 2019, USA). Furthermore, to accurately measure the ionizing dosimeter, the correction factor for temperature and atmospheric pressure was used after confirming that it was 1.0 at a laboratory temperature of 22 °C and 1 atm^33^.

## Results

The produced thin-film shielding paper was manufactured in the same process as the nanofiber mat manufacturing method. On the basis of the report that the absorption effect of radiation is increased when the pattern of nanofibers constructed inside the shielding paper has a certain directionality, the pattern structure of the nanofibers was configured to be the same as that of the morpho butterfly wing structure through the electrospinning process. Through this, the cross-sectional area where radiation and shielding material particles can interact can be enlarged and the density of the shield can be improved.

**Figure 7:**
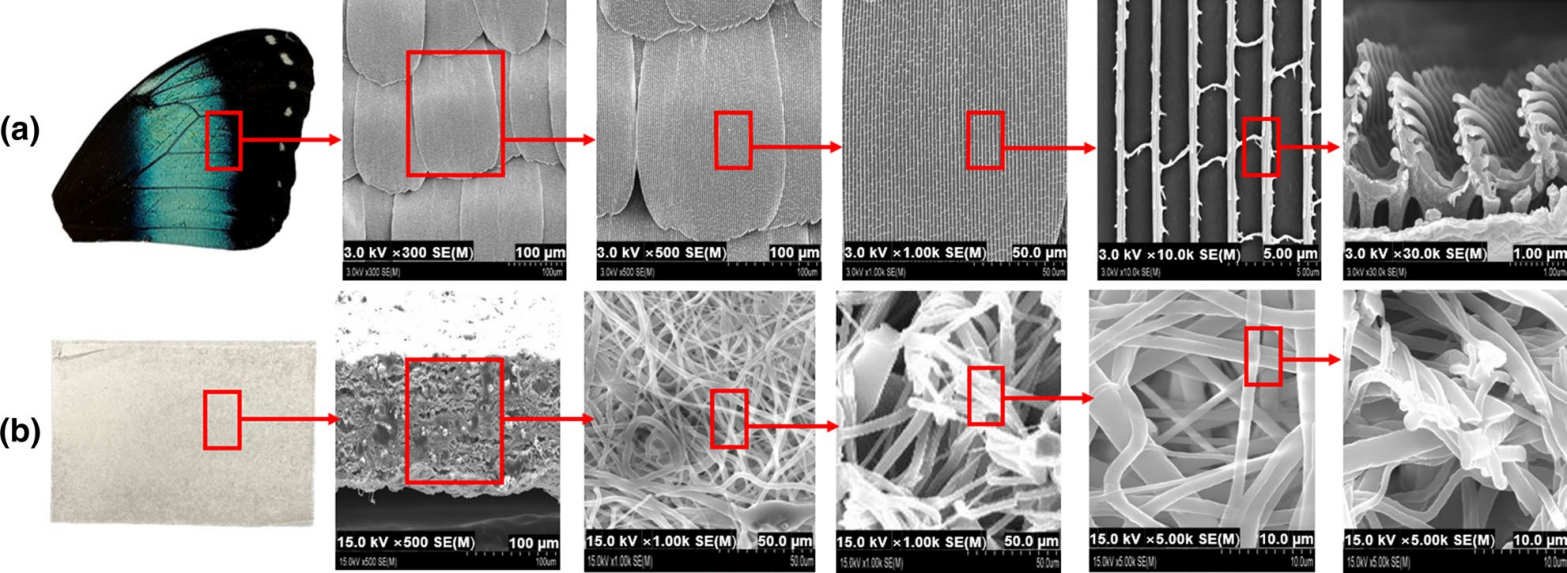
Electron micrographs of (**a**) a morpho butterfly wing and (**b**) the electospun paper.

Figure 7 shows the electron micrographs of the electrospun paper with the same multi-layer structure as a morpho butterfly wing. As shown in Fig. 7, the cross-sectional pattern of the shielding paper was similar to the structure of the morpho butterfly wing. The multi-layer pattern of the shielding paper was spun in a uniform multi-layer structure by controlling the directionality during electrospinning.

**Figure 8:**
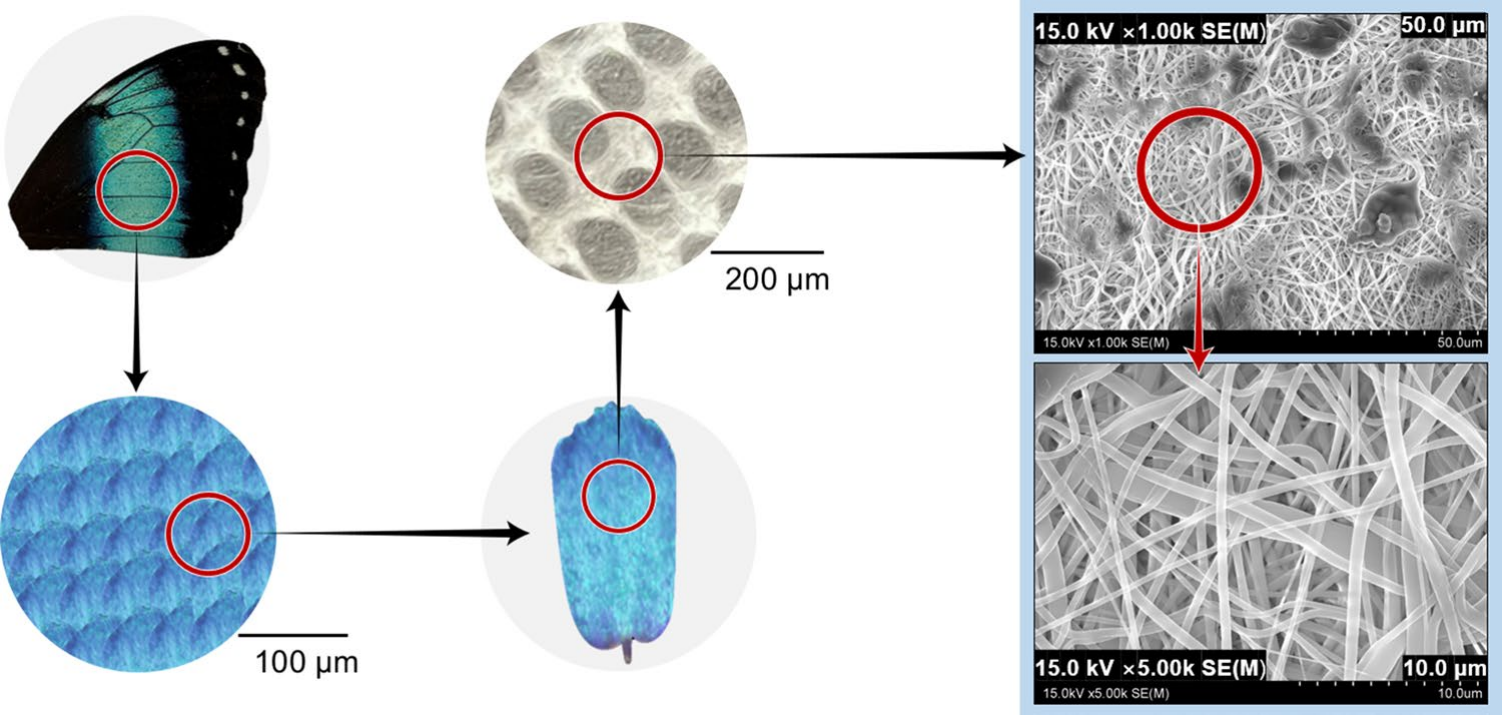
Final microstructure of the electrospun nanofibers.

The microstructure of the shielding paper is shown in Fig. 8. The directionality of the nanofibers was found to intersect with each other. The nanofiber mats prepared before containing tungsten were repeatedly implemented in the form of crossing each other like a woven fabric.

**Figure 9:**
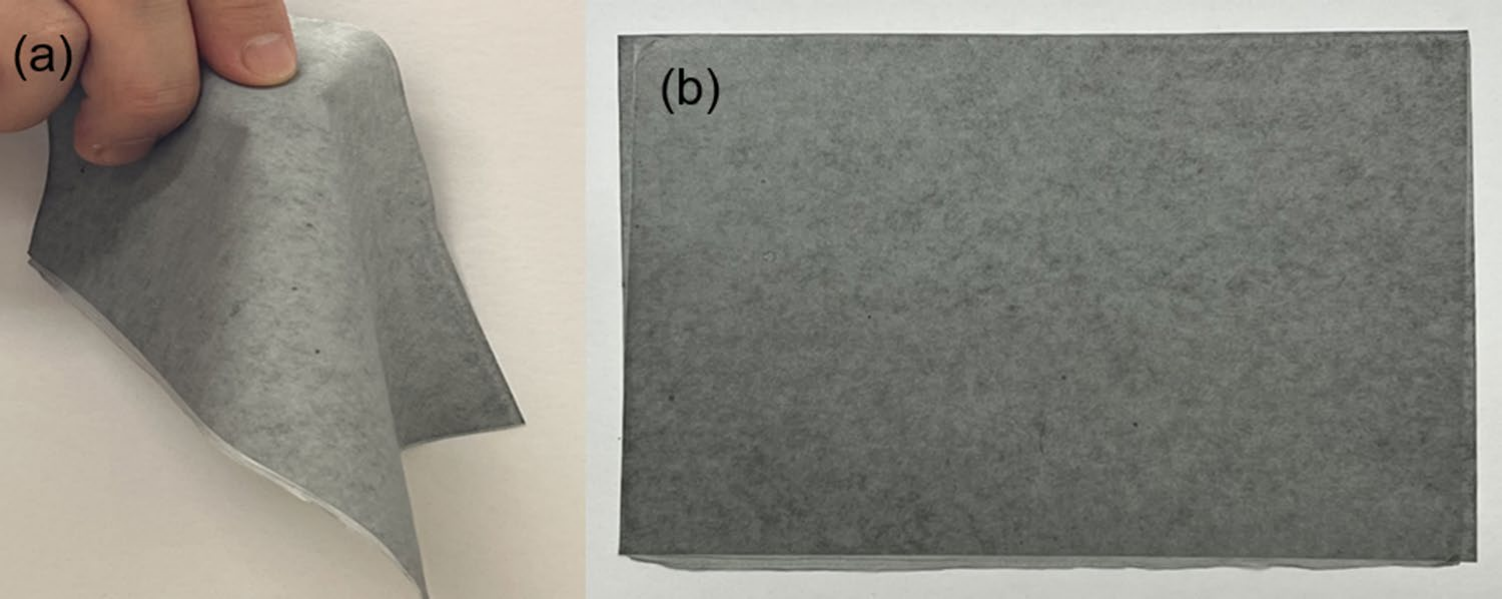
Appearance of the shielding paper (**a**) shows the flexibility of the shielding paper, (**b**) the appearance of the manufactured shielding paper.

The thickness of the flexible shielding paper, as shown in Fig. 9, was 0.1 mm. The physical characteristics of the shielding paper are listed in Table 2.

**Table 2:** Physical characteristics of the shielding paper.

Weight (kg m^−2^)	Tungsten weight (kg m^−2^)	Thickness (mm)	Density (g cm^−3^)	Tensile modulus (MPa)
0.641 ± 0.003	0.401 ± 0.002	0.100 ± 0.003	2.063 ± 0.000	36 ± 0.5

**Figure 10:**
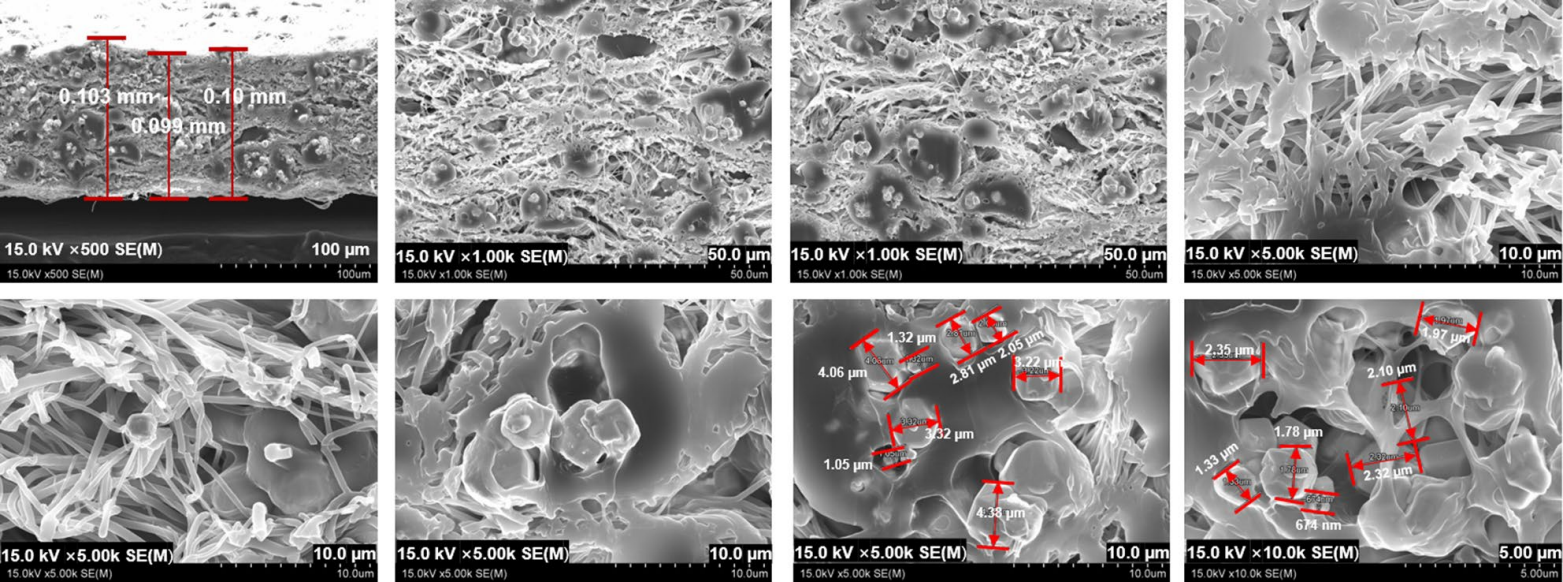
Cross-sectional electron micrographs of the shielding paper.

As for the pattern implemented with nanofibers, in general, the more complex the structure, the more the loss of nanofibers increases, so it can be difficult to implement the pattern in a desired shape. However, in this study, the spinning distance was controlled using the weight of the tungsten metal particles. Figure 10 shows that the tungsten particles were attached to the end of the fiber surface owing to the particle weight, thereby forming a multi-layered shape. When the tungsten particles underwent the thermocompression treatment in the final process, it can be confirmed that the miscibility of nanofibers and tungsten was improved, so that the tungsten particles were uniformly dispersed. The density was as high as 2.463 g cm^−3^, and by unit area it was 0.641 kg m^−2^.

The shielding performance of the manufactured shielding paper was compared with that of a 0.25-mm lead shield. For this, the number of layers in the shielding paper was varied from one to three, and a phantom hand was imaged using X-ray. Table 3 lists the shielding performances of 0.1–0.3 mm standard lead samples (purity 99.9%). For low-energy radiation, 0.3 mmPb showed the highest shielding efficiency of $\ge$ 99%.

**Table 3:** Shielding performance evaluation of standard lead plates.

mmPb	Transmission dose	Effective X-ray energy							
		60 kVp		80 kVp		100 kVp		120 kVp	
		None	Lead	None	Lead	None	Lead	None	Lead
0.1	Dose (mSv)	0.312	0.009	0.854	0.081	1.117	0.136	1.583	0.258
	Shielding rate (%)	–	97.12	–	90.52	–	87.82	–	83.70
0.2	Dose (mSv)	0.312	0.002	0.854	0.043	1.117	0.085	1.583	0.167
	Shielding rate (%)	–	99.36	–	94.96	–	92.39	–	89.45
0.3	Dose (mSv)	0.312	0.001	0.854	0.028	1.117	0.059	1.583	0.124
	Shielding rate (%)	–	99.68	–	96.72	–	94.72	–	91.17

Considering that the lead used in aprons for X-ray shielding in medical institutions is 0.25 mmPb, the shielding performance of the shielding paper developed in this study was compared with that of a 0.25 mm lead plate, and the results are shown in Table 4. Three sheets were stacked to obtain a 0.3 mm thick shielding paper. The shielding performances of the 0.25 mm lead plate and the stacked shielding paper were different by approximately 2% for all effective X-ray energies. Table 5 lists the shielding performance of one-, two-, and three-layered shielding papers. Similar to the previous experiment, the shielding performance of the three-layer shielding paper was equivalent to that of 0.296 mmPb.

**Table 4:** Comparison of the shielding performances of a three-layer shielding paper and 0.25-mm lead plate.

Radiation type	Effective X-ray energy (keV)	Mean of exposure (µR)			Shielding rate (%)	
		Nothing	0.25 mmPb	Shielding paper (0.3 mm)	0.25 mmPb	Shielding paper (0.3 mm)
X-ray	29.2	0.312	0.002	0.008	99.36	97.40
	34.5	0.854	0.039	0.036	95.43	95.80
	52.8	1.212	0.082	0.086	93.23	92.30
	60.3	1.583	0.156	0.157	90.15	90.10

**Table 5:** Comparative evaluation of the shielding performance of the shielding paper with thickness.

Thickness (mm)	Transmission dose	Effective X-ray energy							
		29.2 keV		34.5 keV		52.8 keV		60.3 keV	
		None	Paper	None	Paper	None	Paper	None	Paper
0.1	Dose (mSv)	0.312	0.065	0.854	0.238	1.117	0.368	1.583	0.556
	Shielding rate (%)	–	79.01	–	72.13	–	67.05	–	64.88
	Lead equivalent	–	0.081	–	0.080	–	0.076	–	0.078
0.2	Dose (mSv)	0.312	0.028	0.854	0.115	1.117	0.231	1.583	0.437
	Shielding rate (%)	–	91.02	–	86.50	–	79.30	–	72.40
	Lead equivalent	–	0.183	–	0.182	–	0.172	–	0.162
0.3	Dose (mSv)	0.312	0.008	0.854	0.036	1.117	0.086	1.583	0.157
	Shielding rate (%)	–	97.40	–	95.80	–	92.30	–	90.10
	Lead equivalent	–	0.293	–	0.297	–	0.292	–	0.296

**Figure 11:**
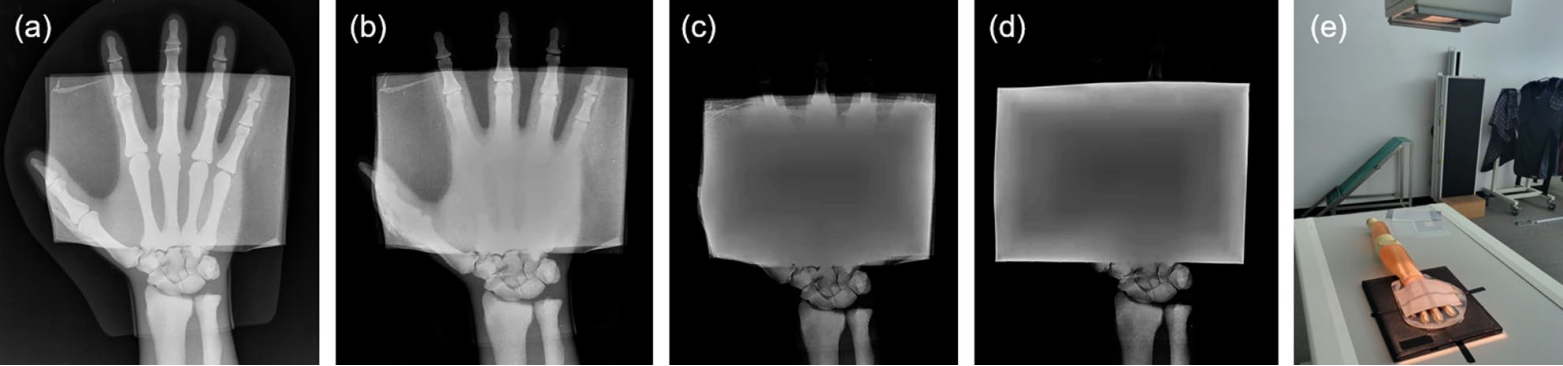
X-ray images of a phantom hand covered with (**a**) one shielding paper, (**b**) two shielding papers, (**c**) three shielding papers, and (**d**) 0.25 mm lead plate. (**e**) Phantom X-ray condition.

To visually verify the shielding effect, X-ray image results of the lead sheet and shielding paper are compared, as shown in Fig. 11, using a hand phantom with the same absorption coefficient as that of the human body. As is apparent from Fig. 11, the shielding performance of the three-layer shielding paper is similar to that of 0.25 mmPb, indicating that it can be used as a shield for medical applications.

## Discussion

Radiation shielding of medical institutions should prioritize ensuring the safety of exposure to patients and medical personnel. However, the shielding product currently provided creates difficulty in user activity owing to its weight. Therefore, a product that is both lightweight and environment-friendly must be developed. Various attempts have been made to increase the density of shielding materials while maintaining the same shielding performance for the same area of the shield^34^. It is challenging to overcome the mass limit because the atomic number of the shielding material must be high. However, the thickness of the shield can be controlled through particle dispersion technology and the manufacturing process, and this can solve the weight issue to some extent.

In this study, a shielding material dispersion technology that maximizes the particle–radiation interaction was investigated. When the dispersed particles interacted with incident radiation or when the transmitted energy was attenuated because of the high density of the shield, the intensity of the incident energy was attenuated^35^. This study was inspired by the structure of morpho butterfly wings and was performed with the expectation that the shielding effect would be higher than that of the existing sheet-type shield if the tungsten particles were dispersed inside the structure. If a composite material, one that mixes shielding material particles with a polymer, is used when manufacturing a shield, there is a limit to the control of the thickness of the shield. The most significant factor for thickness control is the miscibility of the polymer and the shielding material particles. Poor miscibility results in the polymeric and shielding materials aggregating, making it difficult to control the thickness and uniformity of the shield^36^. In this study, electrospinning technology was used as a way to solve these problems, and the shielding effect according to the radiation pattern was confirmed. Therefore, the existing tungsten particle dispersion method was combined with a new technology to reduce the thickness of the shield and improve the shielding effect.

The production of shielding sheets is the most basic process in the manufacture of a shielding apron, and the content of the shielding material is approximately 80–85 wt% when made with a 0.25 mm sheet based on lead equivalent^37^. Additionally, when the content is higher than the aforementioned value, an issue occurs in the tensile strength of the sheet. Therefore, the manufactured thickness is maintained approximately as 0.3–0.5 mm. The weight is approximately 2.80–2.914 kg m^−2^, and the content of the shielding material is generally proportional to the weight of the sheet. In this sheet manufacturing process, uniform dispersion of the shielding material particles is difficult because the particles are non-uniformly arranged during the stirring process of the polymer material and the shielding material. The weight of a single sheet of shielding paper manufactured in this study was 0.641 kg m^−2^, and, when stacked with three sheets, it was 1.923 kg m^−2^. This implies that 1 m^2^ of the shielding paper, required to manufacture one apron, would weigh around 2 kg. Therefore, compared to existing aprons, the weight of an apron, when manufactured with the developed paper, can be reduced by approximately 1 kg and can further improve the mobility of the wearer.

Further reduction in the thickness of shielding clothing can be achieved by using nano-sized shielding particles. However, when manufacturing shielding fibers using nanoparticles, it is inefficient in terms of cost and has limitations in material processing, making mass production difficult^38^. When nano-sized particles of shielding material are mixed with high-density rubber-based materials, they can contain up to 90 wt% of the shielding material. However, there is a limit to reducing the weight of the shield because of the high content of the shielding material. In addition, when the shield is manufactured using a compression process, the thickness of the shield can be reduced, but since the shield is manufactured in the form of a film, there is a problem with flexibility. Therefore, to ensure shielding performance, it is necessary to develop mass production technologies considering the flexibility, tensile strength, weight, and durability of the shield^39^.

In the process presented in this study, tungsten particles and polymer materials were mixed, and electrospinning was performed through a syringe. If this process technology is performed by installing a large number of syringes, all samples can obtain the same shielding effect, so mass production of shields and reproducibility of shielding performance can be secured. In addition, it is effective in terms of economics, as it can use micro-sized shielding material particles rather than nano-size particles.

As mentioned above, the shielding performance of the paper can be controlled by manipulating its thickness and its density can be controlled by changing the temperature and pressure (i.e., the spinning conditions). However, if excessive pressure and temperature are applied to improve the shielding effect, the shield may lose its flexibility. In addition, the content of the shielding material can be increased during the shield manufacturing process, but a delicate technique is required because a difference in shield density occurs depending on the distance at which the spinning solution is spun. In particular, when mass-producing shields, designing these conditions is more important in order to reproduce the same shielding performance.

By controlling the processing conditions a variety of structures suitable for different applications can be produced. For example, the shielding paper can also be used as a radiation-shielding garment against indirect medical radiation (scattering radiation) at a distance of 1.5 m from the radiation source. Furthermore, this material could be used for manufacturing medical products such as surgical gloves^40^. When used for low-dose radiation shielding at less than 100 mSv, the shielding paper could be effective in protecting medical staff and patients^41^. If a shielding material other than tungsten is used, it can contribute to the development of products for cosmic radiation shielding necessary for daily life, such as crew protective clothing and hats for aviation shielding. The thickness and weight of the shield are important factors in shielding cosmic radiation. The results show that the material developed using the proposed process would exhibit effective shielding behavior. Therefore, the production of various shielding clothing that ensures user activity is possible with the process technology presented in this study.

## Conclusion

To reduce the weight of the radiation shield used in medical institutions, a shielding paper was manufactured from nanofibers with a morpho butterfly wing pattern. When the particles of the shielding material are dispersed in a multi-layered pattern, the thickness of the shield can be reduced while increasing the photon collision cross-sectional area, so that the intensity of particle radiation can be effectively attenuated. Compared to the sheet used as the material for the existing apron shield, the weight of the shielding paper manufactured in this study was reduced by approximately 45%. Moreover, the shielding performance of a stack of three sheets of the shielding paper (combined thickness = 0.3 mm) was similar to that obtained by the use of a lead sheet of the same thickness. Therefore, the shielding paper manufactured by the process in this study could be used to develop various lightweight shields and shielding suits for medical institutions.

## Data Availability

All data generated or analysed during this study are included in this published article (and its Supplementary Information files).
